# The Effect of Implementing Advanced Strategies to Improve Immunization Coverage in the Elogbele Health Area

**DOI:** 10.7759/cureus.85346

**Published:** 2025-06-04

**Authors:** Alex Stéphane Ndjip Ndjock, Steve Roland Souga, Elechta Ekow, Michaela Josee Meli, Kristiane Delphine Sunga Yossa, Samar A Amer

**Affiliations:** 1 Association for the Development of Field Epidemiology, Department of Public Health, Edea District Health Services, Edea, CMR; 2 Cameroon Society of Epidemiology (CaSE), Faculty of Health Sciences, University des Montagnes, Bangangte, CMR; 3 Department of Public Health, Edea District Health Services, Edea, CMR; 4 Department of Public Health, Université de Dschang, Dschang, CMR; 5 Department of Public Health, University of Douala, Douala, CMR; 6 Department of Family Medicine, Royal College of General Practitioners, London, GBR; 7 Department of Public Health and Community Medicine, Zagazig University, Zagazig, EGY

**Keywords:** advanced strategy, cameroon, elogbele, immunization coverage, vaccination

## Abstract

Background: Vaccination is a key component of primary healthcare, preventing millions of deaths each year. Therefore, this study aims to explore the impact of advanced vaccination strategies on enhancing immunization coverage in the Elogbele health area from January to May 2023.

Methods: We conducted this retrospective study in the Elogbele health area in the Edea health district, Littoral, Cameroon. We extracted the variables for the number of vaccinated children from the vaccination and tally registers of the area's health facilities and analyzed them using Microsoft Excel 2016 (Microsoft Corporation, Redmond, USA).

Results: The activities carried out in the area led to a clear improvement in the number of vaccinated children with Bacille Calmette-Guérin (BCG) (14 vs. 15), measles rubella vaccine first dose (MR 1) (16 vs. 89), pentavalent vaccine first dose (PENTA 1) (9 vs. 103), and pentavalent vaccine third dose (PENTA 3) (13 vs. 130), as well as in their BCG (2.31% vs. 2.47%), MR 1 (2.82% vs. 15.67%), PENTA 1 (1.58% vs. 18.13%), and PENTA 3 (2.29% vs. 22.89%) vaccination coverage.

Conclusion: This study has demonstrated the importance of implementing advanced strategies and enabled health workers in the Elogbele health area to strengthen their immunization capabilities, improve immunization coverage, and catch up with the number of unimmunized children.

## Introduction

Vaccination is a simple, safe, and effective way of protecting you against harmful diseases, before you come into contact with them. It uses your body’s natural defenses to build resistance to specific infections and makes your immune system stronger. Vaccination has been a significant achievement in public health during the 20th century, serving as the best investment in health, a key element of primary healthcare, an undeniable human right, and the foundation of global health security. It plays an essential role in preventing, reducing transmission, protecting against, and combating outbreaks of infectious diseases, particularly those without medical treatment that can cause serious consequences and even death. Vaccination triggers a reaction in the immune system by training the body's natural defenses to protect it better. Furthermore, it combats antimicrobial resistance [1,2].

Since 1974, the World Health Organization (WHO) has established the Expanded Program on Immunization (EPI). Since EPI's founding, vaccines have saved 154 million lives, 146 million of whom were under 5, including 101 million newborns. Measles immunization saves the most lives, preventing 93.7 million of the 154 million deaths, reducing worldwide newborn mortality by 40%, and African infant mortality by 52%. The vaccine prevents more than five million deaths from diseases like diphtheria, tetanus, and pertussis (DPT), whooping cough, influenza, measles, tuberculosis, poliomyelitis, hepatitis B, yellow fever, meningitis A, and emerging diseases like COVID-19, thereby saving millions of lives [3,4].

In 2006, global immunization coverage stabilized at 80%, indicating that one-fifth of the global birth cohort remains unvaccinated with DPT3. In 2017, the WHO reported that missed opportunities for vaccination (MOVs) in Africa are 96% of children visiting health facilities for care left without receiving the eligible vaccines [4]. During 2023, about 84% of infants worldwide (108 million) received three doses of diphtheria-tetanus-pertussis (DTP3) vaccine, protecting them against infectious diseases that can cause serious illness and disability or be fatal. However, these global figures hide significant disparity among countries of different income strata, with low-income countries lagging behind. In these countries, MOVs have been linked to the national immunization coverage goals, with children who are eligible for national programs not getting all their shots, which makes it harder to reach. In most instances, MOVs result from non-adherence to established norms and procedures in the provision of vaccination services. There is a lot of evidence that the EPI works, but vaccination coverage is still not high enough. Many African countries attribute this MOV to their poor performance in vaccination efforts [5-8].

Adopting high vaccination coverage is recommended as the primary public health measure to manage and stabilize the epidemic patterns of most infectious diseases, with a final goal of establishing herd immunity [3]. Vaccination uptake is influenced by three interrelated factors: (i) Extrinsic factors: External societal and cultural pressures, such as community stigma against vaccines (social influence), misinformation from religious leaders (cultural norms), and peer or family disapproval (group dynamics); (ii) Intrinsic factors: Individual motivations, including fear of side effects (risk perception), personal beliefs about vaccine efficacy (trust), and desire to protect family/community (prosocial motivation); (iii) Structural factors: Systemic barriers, such as distance to health facilities (accessibility), stockouts of vaccines (supply chain gaps), and inflexible clinic hours (service delivery constraints) [9-11].

However, despite enormous progress, vaccination coverage has stagnated in recent years and even fell for the first time in 10 years in 2020. Over the past two years, the COVID-19 pandemic has caused disruption and strain on health systems, resulting in the inability to vaccinate 23 million children in 2020 which is 3.7 million more than what was recorded in 2019 and the highest number since 2009. The World Health Organization (WHO) estimates that yearly, nine million children in the African region lack vital vaccines, and 80% of them live in Nigeria, the Democratic Republic of Congo (DRC), Ethiopia, Angola, Chad, Cameroon, South Africa, Guinea, the United Republic of Tanzania, and Nigeria [12].

Several African countries have reported measles epidemics since January 2020, due to low routine vaccination coverage and delayed vaccination campaigns. In the African region, 10.1 million of the 23 million under- or unvaccinated children worldwide received vaccinations in 2020, up from 9.5 million in 2019. Furthermore, estimates suggest that 21% of infants in the African region have not received the first dose of a DPT vaccine. These "zero-dose" children numbered 7.1 million in 2019 and 7.7 million in 2020. These are children from families and communities who are "likely to lack access to other health and social services and suffer multiple deprivations" [13].

The Elogbele health area is one of 11 in the Edea health district. The Elogbele rural health area comprises four functional health facilities, which are the sixth category in Cameroon's health pyramid, including the Batombe Integrated Health Centre (CSI), a public facility that leads the area, and three private facilities (the EPC Elogbele Health Centre (CS), CS le Secours, and Balm in Gilead Health Centre).

Since the start of 2023, there has been a noticeable decline in immunization performance in this health area, with certain health facilities like the Batombe CSI and the Elogbele CS EPC offering limited immunization services. During the first quarter of 2023 (January, February, and March), there was a noticeable decline in immunization performance. These performances were the lowest in the district in terms of children's vaccination for the tracer antigens Bacille Calmette-Guérin (BCG) (14), Pentavalent vaccine first dose (PENTA 1) (09), Pentavalent vaccine third dose (Penta 3) (13), and RR 1 (16). These very low figures undermine the district's performance in relation to the expected monthly targets. They represent barely 5% of the number of vaccinated children in the whole district, which for the same period was BCG (1142), Penta 1 (876), Penta 3 (655), and RR 1 (809).

Very few studies have been conducted to evaluate the effectiveness of advanced immunization strategies implemented in the Edea health district. This analysis aims to measure the short-term effect of these strategies on childhood immunization coverage between January and May 2023, quantify changes in coverage rates for four tracer vaccines (BCG, PENTA1, PENTA3, and MR1), and evaluate operational outcomes.

## Materials and methods

Study design and setting

We conducted a retrospective comparative study in the Elogbele health area, Edea health district, Littoral, Cameroon, spanning a random sample of health facilities, including CSI Batombe, CS EPC Elogbele, CS le Secours, and Balm in Gilead Health Centre.

Advanced vaccination strategies include mass vaccination, catch-up campaigns, innovative approaches such as vaccination in schools, and the fight against misinformation to increase confidence. These strategies aim to achieve wider vaccination coverage and protect more people against vaccine-preventable diseases.

Data before the implementation of the advanced vaccination strategies in January to March 2023 and after the implementation of the advanced vaccination strategies from April to May 2023 were extracted.

The compressed evaluation timeline (April-May 2023 post-intervention) was necessitated by the urgent need to assess preliminary effectiveness of Cameroon's new immunization acceleration program, synchronization with the national 'Big Catch-Up' initiative timeline following COVID-19 disruptions.

This study included all available immunization records from all four functional health facilities in the Elogbele health area during the study period (January-May 2023), comprising complete vaccination records for 52 children pre-intervention (January-March) and complete records for 337 children post-intervention (April-May).

Pre-intervention baselines were established through record audits, direct observation, and stakeholder verification (findings presented to district health officials for confirmation)

Data collection tool and analysis

The extracted data is secondary data collected from the following sources: vaccination registers, tally books, vaccination coverage monitoring sheets, vaccine temperature monitoring sheets, lost-to-follow-up registers, contingency plans, vaccine order forms, vaccine stock/movement registers, District Health Information Software 2, and post-vaccine adverse event notification forms from health facilities in the Elogbele health area. We utilized laptop computers and other tools.

The data was collected through well-prepared Excel sheets that extracted data before and after the implementation of the advanced vaccination strategies, including the following variables: the number of vaccinated children and the vaccination coverage for each vaccine. Moreover, we used interactive exercises to highlight the impact of advanced strategies on the Elogbele Health Area.

An evaluation grid (form) used for formative supervision of routine immunization indicators, available on the Open Data Kit (ODK) Collect application, was administered to stakeholders to evaluate the interventions.

Data extraction and cleaning utilized standardized Excel (Microsoft 365) templates designed for EPI (Expanded Program on Immunization) monitoring, containing (i) Pre-configured sheets for each vaccine type (BCG, PENTA1/3, MR1) with: Automated coverage percentage calculations ([Doses administered] ÷ [Target population] × 100); Data validation rules to prevent entry errors (date ranges, dose number limits); (ii) Cross-tabulation matrices linking facility registers (data source), DHIS2 export formats (for verification), WHO coverage assessment indicators; (iii) Quality control features: Conditional formatting highlighting outliers (>3SD from mean), VLOOKUP functions to reconcile paper vs digital records, Macros for duplicate detection across facilities

These templates were pretested during December 2022 piloting, showing 98.7% agreement with manual calculations (κ=0.92, p<0.001).

Implementation of the advanced vaccination strategies in the Elogbele Health Area

Health Personnel

To implement advanced strategies, we engaged the following health workers across Elogbele’s four functional facilities, targeting their specific roles in immunization delivery (Table 1).

**Table 1:** Health personnel who participated in vaccinations at the health facilities in the Elogbele Health Area

Health area	Profile	Health Facility
Elogbele	01 Patented Nurse Midwife	Batombe Integrated Health Centre
	01 Nurse	Le Secours Health Centre
	01 Nurse	Cameroon Presbyterian Church Health Centre
	01 Nurse	Balm in Gilead Health Centre

Activities Carried Out

The following activities were carried out during the aforementioned working sessions: The activities included enhancing the understanding of vaccination practices among the staff. The team is currently being briefed on the development of an advanced strategy plan. The organization is committed to supporting vaccine conservation efforts by providing essential data management tools, including vaccination registers, tally books, and vaccination cards. Vaccine supply: The community is receiving support for the implementation of advanced strategies.

As part of group work sessions, these health workers were trained in the rigorous application of vaccination protocols and the use of DHIS2 dashboards to monitor coverage in real time. Furthermore, targeted community awareness campaigns were implemented. The primary objective of these initiatives was to enhance the vaccination rate of children aged 0-59 months (a demographic segment of the Cameroon EPI target population) residing in the Elogbele health area.

Three-day interactive workshops were organized, combining theoretical training on EPI protocols according to the 2022 WHO guidelines, practical vaccine simulation exercises, and role-playing games to manage vaccine hesitancy.

This process includes gap analysis using the WHO Immunization Barriers Assessment tool, as well as data review in the Situation Room. Personalized micro-plans were created for each health center, with an evaluation after each campaign.

Key interventions to optimize vaccine management included the distribution of cold chain temperature sensors (Fridge Tag 2), the implementation of stock monitoring dashboards via DHIS2, and the development of emergency buffer stocks at the health zone level.

Door-to-door campaigns targeting areas with low coverage were also carried out, as well as dialogues with local leaders (churches, schools, markets).

Ethical considerations

This study adhered to the Declaration of Helsinki. We went through a series of steps to obtain the various authorizations needed to collect data in the Edea health district.

## Results

The activities carried out are impacted by the area's performance. The activities (healthcare worker training, community mobilization, data-driven outreach, cold chain management) were carried out in the Elogbele health area as part of this project. All of this contributed positively to increasing the quantity of vaccines administered to the target population, resulting in better immunization coverage in the area (Table 2).

**Table 2:** Details of the impact of advanced strategies in the Elogbele health area

Before	After
Insufficient knowledge among stakeholders about vaccination in practice	Improved knowledge of vaccination in practice among stakeholders
Lack of advanced strategy planning in the Elogbele health area	Availability of advanced strategy planning in the Elogbele health area
Failure to implement advanced vaccination strategies	Implementing advanced vaccination strategies
No search for the lost to follow up to vaccination	Planning and searching for the lost to follow up to vaccination
Low vaccination coverage	Increase in vaccination coverage for all antigens
No vaccination services offered in some health facilities	Batombe Integrated Health Centre
Lack of planning and implementation of awareness-raising sessions in the community	Planning and carrying out mass awareness campaigns in the community on the importance of vaccination
No use of community health workers during vaccination sessions	Use of community health workers during vaccination sessions
Poor vaccine management	Improved management (conservation) of vaccines
Failure to draw up order forms	Preparation of purchase orders and timely transmission to the district
Lack of advanced strategy planning for the area	Availability of advanced strategy planning

Number of vaccinated children by tracer antigen

**Figure 1:**
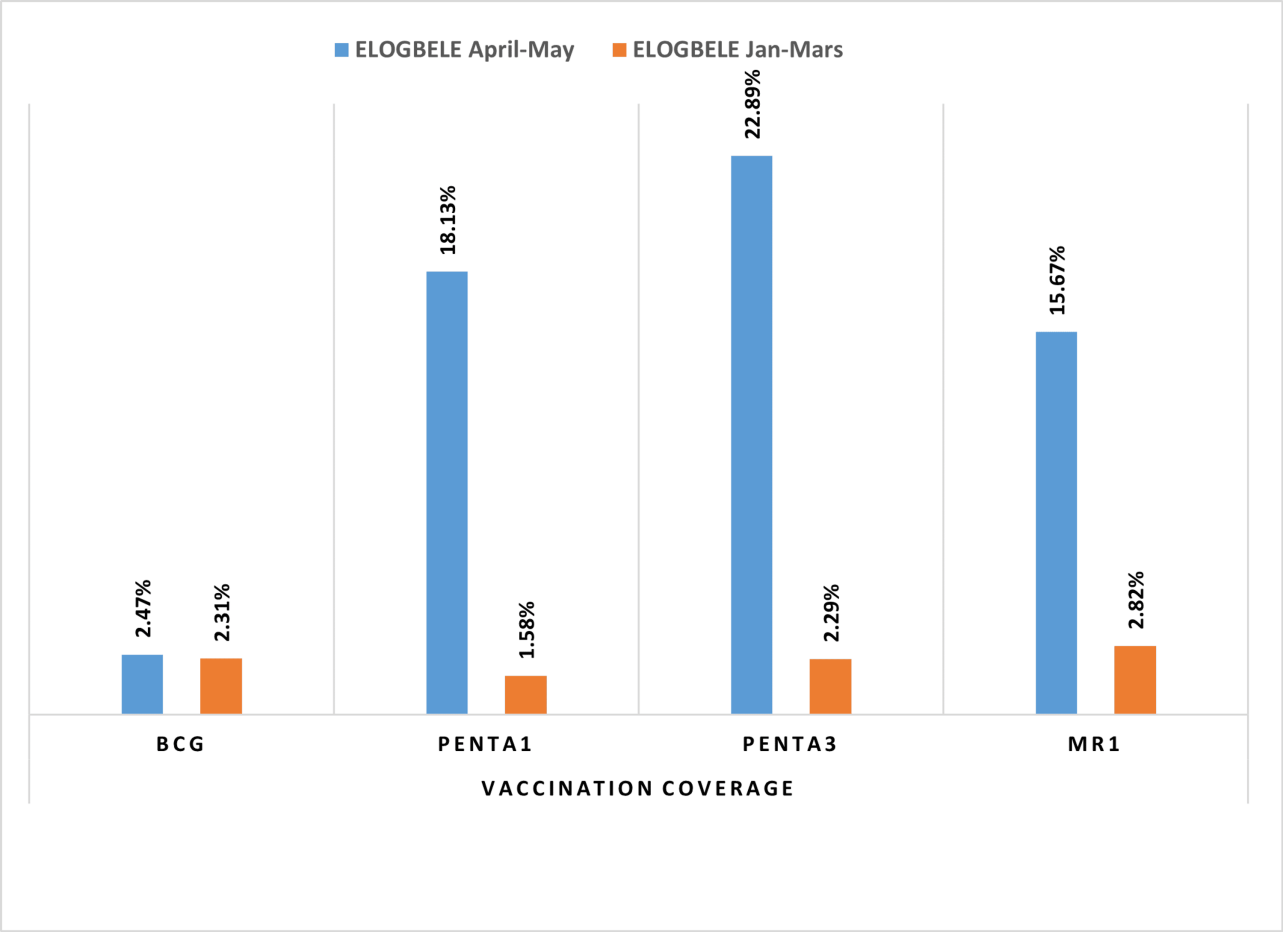
Vaccination coverage per antigen in the Elogbele health area for the January-March and April-May periods

The implementation of immunization activities (healthcare worker training, community mobilization, data-driven outreach, cold chain management) in line with the recommendations led to a significant increase in the number of vaccinated children with tracer antigens during the post-intervention period (April-May) compared with the pre-intervention period (January-March). In the Elogbele health area, the number of vaccinated children rose from 52 to 337, an increase of 285 children (548%). With regard to the number of vaccinated children by antigen, the greatest difference between the number of vaccinated children before and after the intervention was observed in PENTA 3, where the number of vaccinated children rose from 13 to 130, i.e., 117 more children were vaccinated. Conversely, over the same period, BCG vaccination increased from 14 to 15 children, i.e., one more child vaccinated (Figure 1).

Vaccination coverage per tracer antigen

**Figure 2:**
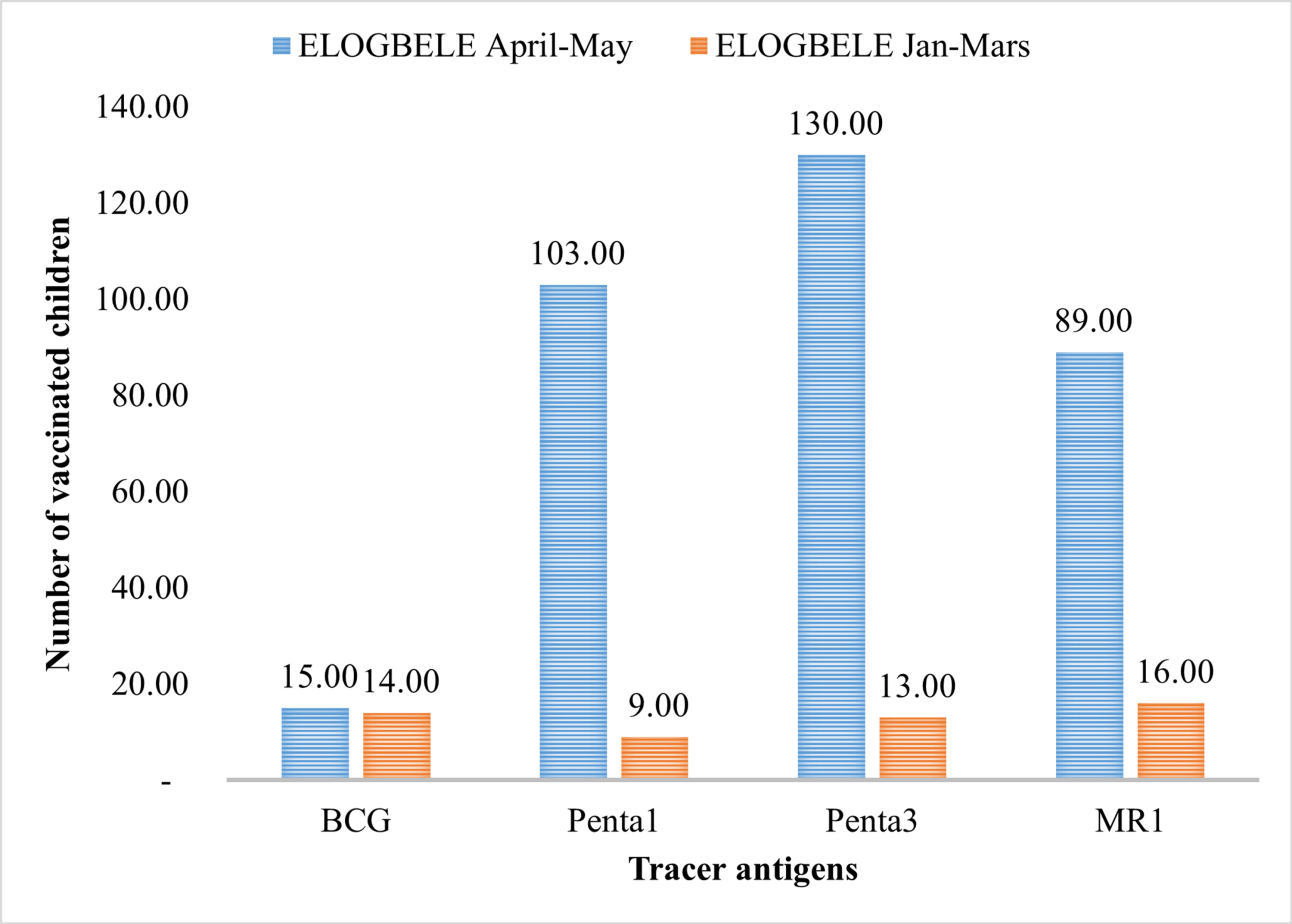
Number of people vaccinated per tracer antigen in the Elogbele health area for the periods January-March/April-May

The vaccination coverage per antigen was used to evaluate the effectiveness of the routine vaccination system in the health area before (January to March 2023) and after (April to May 2023) the introduction of the advanced vaccination strategies. Overall, we observed an improvement in vaccination coverage for each antigen, albeit with significant variations depending on the specific antigen. PENTA 3 coverage improved the most, rising from 2.29% to 22.89%, an improvement of 20.6%, while BCG coverage improved by 0.16%, from 2.31% to 2.47% (Figure 2).

## Discussion

The study's results reported that the implementation of the advanced strategies had a significant impact on improving immunization coverage per antigen and the number of vaccinated children by tracer antigen in the Elogbele health area. Other studies conducted in Cameroon, Ethiopia, Ghana, India, and Kenya have also yielded similar results [13-15].

In April 2023, Ethiopia began implementing advanced vaccination strategies, aligning with the global 2023-2024 EPI vaccination milestones that have had frequent updates since their launch in 1974, 1979, 1982, 1984, 1990, 1999, 2000, 2017, and till now. The timeline spans from 2020 to the present. The global milestone is the Immunization Agenda 2030 (IA2030), which was set up to ensure universal access to vaccines, strengthen healthcare systems, and support universal health coverage. From 2020 to 2023, the milestone is COVID-19 Vaccines Global Access (COVAX) to accelerate the development, production, and equitable distribution of COVID-19 vaccines. Finally, in 2023-2024, the Big Catch-Up initiative focuses on restoring immunization coverage to pre-COVID-19 levels and strengthening routine immunization systems to achieve 2030 targets [16,17].

There was a significant increase in the number of children vaccinated with tracer antigen. In the post-intervention period, there were 337 vaccinated children in the area, representing a 548% increase from the pre-intervention period shown in Figure 2, and the coverage of immunizations by antigen in the eligible health area is due to the implementation of an advanced vaccination strategy, which includes multiple activities, as explained in the methodology section and Tables 1, 2. In particular, community-oriented activities are more intense and better coordinated, leading to a change in health behavior over a longer term, which explains the observed trend. Added to this is the fact that advanced strategies reach the poorest and most marginalized communities due to a lack of resources, building trust through regular interaction, and the supportive activities of community mobilizers drawn from the communities they serve [13,18-20].

Regarding the type of vaccination, BCG had the smallest increase in the number of vaccinated children, adding only one child, or 7% more, compared to PENTA 3, which added 117 children, or 900% more. While the overall increase in vaccinated children aligns with a Nigerian study that saw a 24% increase in vaccinated children, MMR1 showed the most notable increase with 27% more vaccinated children, whereas PENTA 1 showed the least notable increase with only 4% [21]. There may have been differences between these studies because they looked at different study periods and populations. However, the fact that they both increased the number of vaccinated children may be because similar EPI strategies involved and mobilized communities more during the same time periods [22].

The BCG vaccine for tuberculosis had the smallest increase in the number of vaccinated children, and BCG coverage improved by 0.16%. The projected increase is the lowest because the BCG was administered according to the WHO at birth or within the first 28 days of life in most healthcare settings, resulting in the highest coverage rate worldwide. The timing of BCG vaccination is crucial for both obtaining opportune protection and serving as an indicator of non-adherence [23].

Despite the variations observed, all the tracer antigens showed an improvement in their respective vaccine coverage. The highest increase in the number of vaccination doses and coverage was for PENTA 3, which added 117 children, or 900% more, with a coverage value of 20.6%. In contrast, an Indian study showed an increase in all marker antigens but differed from our results in revealing that the highest increase in coverage was achieved by PENTA 1 with a 5.9% improvement in coverage and the lowest by PENTA 3 with 3.1% [9]. All of this demonstrates the need to develop and implement context-specific strategies to close vaccination gaps by catching up missed children, prioritizing essential health services, and strengthening immunization programs to prevent outbreaks [24].

The study's strengths lie in its assessment of the early implemented steps from April to May 2023, the Health Development Plan 2022-2025, the National Development Strategy (SND30), and the Health Sector Strategy (SSS 2020-2030), which demonstrate Cameroon's commitment to enhancing the population's access to necessary and specialized health care. The SND30 and SSS 2020-2030 aim to improve health and well-being in line with Sustainable Development Goal 3 by enhancing the capacity of health districts to deliver primary health care and improve health service quality. These activities and studies will help stakeholders and decision-makers meet the varied health needs of the community [25]. This includes improving access to quality care and strengthening local resources to provide effective services, especially for vaccinations. 

Given that Cameroon's EPI already provides free vaccines, our approach has prioritized optimizing existing systems rather than building new facilities or changing cost structures. The main operational strategies involve improving access to healthcare by extending the opening hours of existing facilities to include evenings and weekends and by deploying mobile teams to remote villages on market days. This eliminates obstacles related to distance. Other strategies include using geo-mapping data to identify households with unvaccinated children and organizing discussions about vaccination in churches and schools to take advantage of existing social networks and facilitate acceptance. Similarly, local resources need to be strengthened by providing solar refrigerators to stabilize the cold chain and ongoing training for healthcare staff on practical immunization. Similarly, providing financial resources to support advanced strategies will motivate health workers and help us to reach every child, thereby achieving optimal immunization coverage.

Therefore, we recommend the following: strengthening the knowledge of the focal points of the expanded program on immunization in the health facilities of the Elogbele health area on immunization in practice; planning and implementing advanced strategies in the Elogbele health area and the community; plan and seeking out those lost for follow-up vaccinations; planning and carrying out mass awareness campaigns in the community on the importance of vaccination and adherence to the vaccination schedule; using community health workers during vaccination sessions; finally, improving vaccination storage and management.

In order to systematically identify and recover lost children, it is important to set up a multi-channel monitoring strategy adapted to the local context of Elogbele, such as monthly cross-checks between the immunization registers of health facilities and the birth registers of traditional birth attendants, in order to flag up children who need to receive doses of vaccine. Initial home visits are also essential in this rural environment. Voice reminders could be added to these visits. Awareness campaigns aimed at religious, traditional, and administrative authorities could encourage community involvement.

The study is limited by being a retrospective study that utilizes secondary data that may affect the quality of the data. The study's focus on all four health facilities within the Elogbele health area (rather than sampling) ensures complete representation of this microsystem but inherently limits direct extrapolation to other contexts. This study shows short-term progress, but the two-month post-intervention period does not allow us to assess the sustainability of the results, the influence of seasonal variations or the retention of skills by healthcare professionals. These constraints, which are typical of rapid evaluations in public health, will be investigated further during the 12-month follow-up.

## Conclusions

This study has demonstrated the importance of implementing advanced strategies and enabled health workers in the Elogbele health area to strengthen their immunization capabilities, improve immunization coverage, and catch up with the number of unimmunized children. It emerged that most of the objectives had been achieved, which is a satisfactory achievement. This project has enabled health workers in the health area to strengthen their knowledge, attitudes, and practices regarding immunization, to improve immunization coverage in the health area, and to catch up with unvaccinated children. Despite restrictions linked to time constraints, constant and ongoing efforts are being made to achieve the target. 
